# Multimodal dynamic retinal vessel analysis offers new insights in microvascular defects in Fabry’s disease

**DOI:** 10.1038/s41598-025-03020-9

**Published:** 2025-06-06

**Authors:** Abdelrahman Assaf, Konstantin Kotliar, Arno Schmidt-Trucksäss, Ines Lanzl

**Affiliations:** 1 Chiemsee Augentagesklinik, Prien am Chiemsee, Germany; 2 Department of Medical Engineering and Technomathematics, FH Aachen University of Applied Sciences, Campus Juelich, Juelich, Germany; 3 Department of Exercise and Health Sciences, University of Basel, Basel, Switzerland; 4 Rechts der Isar Hospital , Technical University of Munich, Munich, Germany; 5 Eye clinic, Rechts der Isar Hospital , Technical University of Munich, Munich, Germany

**Keywords:** Fabry’s disease (Morbus Fabry), Dynamic retinal vessel analysis, Flickering light, Retinal microcirculation, Smooth muscle, Potassium channel, Disease genetics, Metabolic disorders

## Abstract

Fabry’s Disease (FD) is one of many disorders that result in altered vascular function. With the Dynamic Vessel Analyzer (DVA, IMEDOS Systems), retinal vessels can be recorded non-invasively in real time and dynamic responses to stimuli that affect vessel diameter can be directly visualized. The data obtained can be further mathematically evaluated in terms of dynamic time- and location-dependent vessel behavior. This principle of multimodal analysis of dynamic retinal vascular behavior has been applied to demonstrate specific structural and functional retinal microvascular changes in Fabry’s disease. The retinal vascular response was examined with DVA in 10 patients with FD, 4 women, 6 men including 1 child aged 42.5 (34.3–57.3) years (median (1st quartile–3rd quartile)) and in 10 age- and sex-matched healthy participants. The vessel width of an arterial and a venous retinal vessel segment of ~ 1 mm in length was examined in all participants. After 50 s of baseline observation, a monochromatic rectangular luminance flicker (530–600 nm; of 12.5 Hz frequency) was applied three times for 20 s. The vascular response to flicker light was analyzed. Using mathematical signal analysis, the longitudinal microstructure of retinal vessels was assessed and unstimulated vessel wall oscillations were characterized. Group comparisons were performed exploratively using Mann–Whitney-U test. The entire patient group showed no significant difference in response to the flicker light compared to the control group. After dividing the patient group by sex, male patients with FD showed significantly larger arterial dilatation of 6.3 (5.6–7.9)% compared to age-matched controls: 3.3 (2.5–4.1)%, *p* < 0.01. In contrast, female patients showed a reduced arterial response of 3.4 (3.1–3.7)% compared to age-matched controls: 5.4 (4.0–6.7)%, *p* = 0.1. When analyzing the periodicity of the spontaneous unstimulated vessel wall modulation, female Fabry patients had a higher and less scattered periodicity of vasomotions in arteries and veins, while male patients showed lower periodicity of vasomotions in arteries with more scattered periods and longer more scattered periods in veins, compared to age-matched controls. The cardiac rhythm was more aperiodic and less pronounced in male patients. In addition, the longitudinal microstructure of retinal arteries and veins in FD was structurally and functionally altered. This was particularly notable in veins at all stages of the vascular reaction and in arteries at all stages except the baseline by more pronounced waves with periods of ~ 50–100 µm. Multimodal dynamic retinal vessel analysis conveys information about different aspects of vascular regulatory potential. The flicker provoked retinal arterial response is more pronounced in male patients with FD. This reaction is nitric oxide (NO) mediated and conveyed by the vascular endothelial cells. With an altered baseline smooth muscle activity in vessel walls subsequent reactions to vascular stimuli such as flicker light for the retina may be more pronounced in the imbalanced system of male Fabry patients. Spontaneous retinal vessel oscillations are altered in Fabry’s disease. These alterations are more dramatic in male patients, and are especially characterized with less periodic vessel behavior over the large frequency range. This is a result of altered smooth muscle reaction potentially due to disease mediated altered potassium channel activity. Both arterial and venous retinal vessel walls in FD show peculiar microstructural changes. The characteristic microirregularities of retinal vessels are of functional nature in arteries and rather of structural nature in veins. The DVA examination with multimodal retinal vessel analysis represents a practical possibility to investigate central microvascular status of Fabry’s disease in more detail and might elucidate the effect of potential therapeutic interventions.

## Introduction

In 1898, Anderson and Fabry both published descriptions of patients with a skin disorder characterized by red and purple lesions on the skin^1,2^. Initially, Fabry’s disease or Morbus Fabry or Anderson-Fabry’s disease was referred to as “Angiokeratoma Corporis Diffusum” and was thought to be primarily a dermatological condition. It was described in several case reports^3,4^. Fabry’s disease is a rare, inherited X-linked lysosomal storage disorder^5^. This deficiency is caused by abnormalities in the alpha-galactosidase A gene, which is responsible for breaking down certain fats known as globotriaosylceramides (Gb3)^6^. The accumulation of glycosphingolipids can lead to serious and progressive damage to the kidneys, heart, and other organs, as well as symptoms such as pain, gastrointestinal problems, transient ischemic attacks and strokes. Symptoms of Fabry’s disease usually begin to appear in childhood or adolescence, and can vary widely from one individual to another. The treatment of Fabry’s disease includes enzyme replacement therapy to manage symptoms and prevent organ damage.

Cerebrovascular disease, including stroke, is a common complication of Fabry’s disease. Studies have shown that the incidence of stroke is significantly higher in young men and women with Fabry’s disease compared to the general population. A Fabry Registry study found that the prevalence of stroke was almost 12 times higher in young men and 10 times higher in young women with Fabry’s disease compared to the general population^7^. Symptoms are usually more severe in male patients.

The mechanism of stroke in Fabry’s disease is not fully understood. In the meantime, various treatment modalities, such as antiplatelets, lipid-lowering agents, and inhibitors of the renin–angiotensin–aldosterone system, have been shown to be effective in mitigating vascular dysfunction and reducing the risk of stroke in patients with Fabry’s disease^8^. There are several hypotheses about the mechanisms that contribute to the vascular dysfunction observed in Fabry’s disease. These include^9^:

- Endothelial dysfunction has been linked to the development of vascular problems, such as stroke, in Fabry’s disease.
- Cerebral hyperperfusion: Fabry’s disease has been linked to increased blood flow to the brain, which may contribute to the risk of stroke.
- Pro-thrombotic state: Fabry’s disease has been associated with an increased risk of blood clot formation, which can lead to blockages in blood vessels and increase the risk of stroke.
- Higher synthesis of reactive oxygen species: Fabry’s disease has been linked to increased production of reactive oxygen species, which are highly reactive molecules that can damage cells and tissues. This may contribute to the development of vascular dysfunction in Fabry’s disease.
- Accumulation of sphingolipids in vascular tissues especially in vascular endothelial cells, contributing to the development of vascular dysfunction, which can subsequently lead to stroke and other complications in Fabry’s disease.

**Fig. 1:**
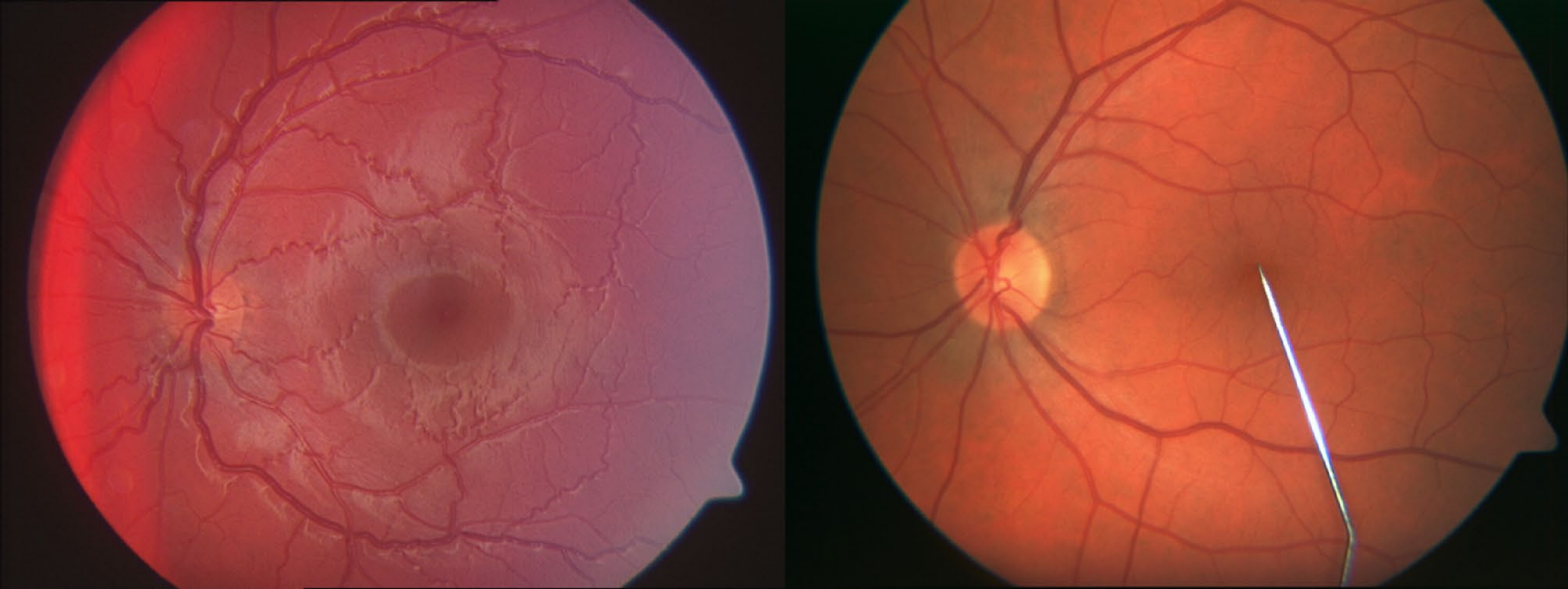
Retinal images of a Fabry patient (left panel) and a healthy volunteer (right panel). Note peculiar tortuous structure of retinal vessels of the patient.

Retinal vessel changes in patients with Fabry’s disease are often subtle and may not be readily detectable during routine clinical examinations, while in some patients, the peculiar tortuous pattern of retinal vessels is clearly visible (Fig. 1). However, advanced techniques such as dynamic retinal vessel analysis offer a more sensitive approach to identify microvascular alterations, which could serve as early biomarkers for cerebral vascular dysfunction associated with the disease^10,11^. Early diagnosis and treatment can prevent long-term complications and improve patient outcomes. To quantify vascular dysfunction accurately, it is essential to observe the blood vessels directly, examining both their structure and function. Retinal blood vessels, which share structural and functional similarities with cerebral microvessels, provide an ideal opportunity for this, as they can be examined non-invasively using modern optical imaging methods. In this study, we utilized dynamic retinal vessel analysis to assess central microcirculatory function in Fabry’s disease.

Using the Dynamic Vessel Analyzer (DVA, IMEDOS Systems), it is possible to non-invasively assess changes in the diameter of retinal vessels at the fundus of the eye in real time, thus enabling an analysis of their dynamic behavior^12,13^. Dynamic retinal vessel analysis is a non-invasive technique that employs specialized imaging equipment to capture detailed sequences of images of retinal blood vessels. These images are subsequently analyzed using computer software to extract various quantitative measures of retinal blood vessel dynamics. These measures relate, for example, to vessel diameter responses to functional stimulation^14,15^, or characteristic frequencies of vessel wall oscillations^16,17^, and microirregularities of the internal vessel wall^18^. Dynamic retinal vessel analysis has several advantages over other imaging techniques of retinal vasculature, such as fluorescein angiography, being quick, painless, and not requiring the use of contrast agents. The abnormalities of retinal vessel behavior measured with DVA can be indicative of the presence and severity of the disease with systemic vascular involvement and may be used to guide treatment decisions. Dynamic retinal vessel analysis has also been shown to be useful in the detection and management of several eye conditions, such as diabetic retinopathy, glaucoma, and age-related macular degeneration^19–21^.

It is noteworthy that retinal and cerebral vasculature are unique in the body since both lack adrenergic neuronal input and vessel width regulation is only performed by local effectors in both. Therefore, retinal vessel behavior represents a window to the brain and its specific vasculature. The purpose of this pilot study was to evaluate pathologic aspects of dynamic retinal vessel behavior in Fabry patients using multimodal retinal vessel analysis, which involves the assessment of different aspects of microvascular function.

## Patients and methods

### Recruitment of patients, ethics statement, inclusion and exclusion criteria

Patients and controls from the Department of Ophthalmology at Technische Universität München were enrolled in this prospective pilot study. Ten patients with Fabry’s disease, 42.5 (34.3–57.3) years old [median (1st quartile–3rd quartile)], 4 women, 6 men including one child and 10 age- and sex-matched medically healthy individuals of the control group were investigated. Only one randomly chosen eye of each participant was included. Slit-lamp biomicroscopy, indirect ophthalmoscopy and fundus photography were performed in each patient.

The inclusion criteria of the Fabry’s disease group was diagnosed Fabry’s disease. All Fabry disease diagnoses were confirmed through genetic testing and enzymatic assays consistent with standard diagnostic criteria. The male patients in the Fabry group were undergoing enzyme replacement therapy, while the female patients were not. None of the Fabry patients were affected by eye disease. Blood pressure was in the normal range in all Fabry patients, which is common in this disease: mean arterial pressure amounted to 94.4 (91.8–102.5) mmHg in the Fabry group and to: 96.3 (90.5–99.0) mmHg in the control group (*p* = 0.67). One male patient was taking candesartan. In addition, the Fabry patients in the study had no evidence of renal insufficiency and were not on dialysis, which might have introduced variability related to disease progression. All Fabry patients were integrated into normal school or work life, reflecting stable disease management.

Ten medically healthy controls were recruited from the Department of Preventive Sports Medicine at Technische Universität München. This control group consisted of healthy individuals with no known systemic diseases or conditions. Their health status was confirmed through a comprehensive internal medicine consultation to ensure the absence of any underlying systemic illness. The validation criteria were: absence of any systemic and eye disease; absence of any systemic therapy; body mass index 19–28 kg⁄m^2^; systemic blood pressure RR_syst_ < 135 mmHg, RR_diast_ < 90 mmHg; blood glucose < 110 mg⁄dl; low-density lipoprotein (LDL) cholesterol < 190 mg⁄dl; high density lipoprotein (HDL) cholesterol > 35 mg⁄dl. Additionally, these individuals were not taking any medications. To enhance the validity of comparisons, the Fabry cohort and the control group were carefully matched based on age and sex: 42.5 (34.0–57.0) years old, 4 female and 6 male individuals in the control group.

Informed consent was obtained from all patients enrolled in the study. The study protocol was reviewed by the ethics committee of Medical Faculty (Klinikum rechts der Isar) at Technische Universität München. All procedures adhered to the tenets of the Declaration of Helsinki.

### Dynamic retinal vessel analysis assessment

Thirty minutes after pupil dilation with three consecutive drops of topical tropicamide (Mydriaticum Stulln; Pharma Stulln Ltd, Stulln, Germany) applied 10 min apart, continuous measurement of retinal arterial and venous diameter was performed using DVA^21^. The properties of DVA and its measurement principles have been described extensively before^12,22,23^. Briefly, the device allows direct non-invasive online assessment of retinal vessel diameters, depending on time and location along the vessel. It performs optical imaging of the retina and consists of a fundus camera with adjusted CCD camera, that produces a film of the retina and transfers the video signal to a computer. The key principle for detection by DVA is that erythrocytes within the vessels absorb light (most effectively at wavelengths of 400–620 nm)^21^, whereas the retinal layers reflect light. This difference in reflection allows DVA to identify the erythrocyte columns and define them as vessel diameters. Using imaging processing analysis, diameters of retinal vessels are assessed along the chosen vessel segment over time. Thus, in this study ‘vessel diameter’ refers to the erythrocyte column as measured by DVA^24^. According to the manufacturer, DVA assesses retinal vessel diameters with temporal resolution of 40 ms and measurement resolution less than 1 µm. Systematic error of non-linearity S ≤ 1.6%^24^. Several independent studies have shown high reproducibility of DVA^23–27^. For example, short-term reproducibility (2 h between measurements) was reported with an intraclass correlation coefficient of 0.98 for venous and 0.96 for arterial measurements^24^.

The time-dependent retinal vascular reaction to monochromatic rectangular luminance flicker (530–600 nm; 12.5 Hz; 20 s) was measured using DVA in each study participant. The standard locations for analysis were upper temporal retinal artery and vein 1–2 optic nerve head diameters away from the optic nerve head rim. Arterial and venous vascular response to flicker light was analyzed in retinal vessel sections ~ 1 mm long. We used a standard 350 s measurement protocol as described in detail elsewhere^12,23^. It included 50 s of baseline recordings followed by 3 consecutive periods of 20 s flicker stimulation. In addition, mathematical signal analysis was applied to characterize the unstimulated vessel wall oscillations and longitudinal microstructure of retinal vessels.

In this study, we referred the term ‘multimodal dynamic retinal vessel analysis’ to the integration of different types of vascular assessments, including baseline vessel diameter, flicker-induced dilation and constriction responses, as well as spontaneous vasomotor oscillations and assessment of spatial arterial blood column diameter change in order to comprehensive characterize retinal microvascular function.

### Examination of the arterial and venous response to flicker light.

The raw numerical data generated from the DVA was exported to an Excel template (MS Excel 2016; Microsoft, Redmond, USA) with macros to allow further filtering, processing and analysis of the data. The DVA software allows data to be exported in a summarized form with one data point calculated per second. We extracted this data from all three flicker curves and generated median values for each 1 s time point, allowing us to construct an average summary curve of a cycle consisting of 30 s baseline, 20 s flicker stimulation and 80 s rest. We then smoothed the average curve by calculating a running median with a window of 4 s as described more in detail before^12,28^. A pilot exploration of representative time courses in the groups gave us a clue for the main outcome parameters. The following parameters were measured:

- Absolute vessel diameter, MU (1 MU corresponds to 1 µm in Gullstrand’s eye model) was calculated individually as a median value during final 30 s before the first flickering;
- Mean maximal vessel dilation in response to flicker, % to baseline, was calculated as the absolute maximum of the smoothed average curve;
- Mean maximal arterial constriction following the flicker cessation, % to baseline, was calculated as the absolute minimum of the smoothed average curve;
- Arterial peak value (max. constriction + max. dilation), % to baseline.

Representative time courses of vessel diameter changes in each group were plotted as a median of all smoothed individual temporal responses of the group^12^. The median time courses show peculiarities of the dynamic behavior of vessel diameter of a group with the following limitation: some calculated average values of introduced parameters may not exactly match a corresponding value on the curve because of the method of calculation.

### Assessment of oscillatory temporal changes of retinal vessel diameter

Oscillatory temporal changes of retinal vessel diameters were assessed and were evaluated using mathematical signal analysis as described previously in detail^16,29^. A temporal sequence of 40 s was chosen within the DVA baseline recording with temporal resolution of 40 ms. A template with MS Excel macros was developed to filter, process, analyze and display the data. An application created in MATLAB (MathWorks, Natick, USA) closed gaps in the signal with spline interpolation, normalized the signal, performed Fourier analysis, and evaluated the chosen parameters. To analyze the frequency distribution within the temporal sequence, we performed autocorrelational analysis and derived some parameters of the autocorrelation functions of the participants. In simplified terms, we built and mathematically analyzed the autocorrelograms of the temporal sequences by automatically detecting local maxima/minima along the curve:

- For the high-frequency (HF) oscillation components (pulsations), searching within a moving frame of 8 previous and 8 subsequent values (corresponds to ± 0.32 s), thus trying to reveal heart-frequency pulsations, as well as,
- For the low-frequency (LF) oscillation components (vasomotions), searching within a larger moving frame of 40 previous and 40 subsequent values (corresponding to ± 1.6 s), thus trying to reveal vasomotions—low-frequency modulations of the vessel wall.

Parameters of the autocorrelation of the temporal sequences were derived for HF and LF vessel oscillations^16,29^:

- *Mean arterial and venous period at HF and LF.* The mean period of the oscillations of the autocorrelation function corresponds to the average period of an analyzed signal. It was calculated as the median of all time intervals between corresponding local maxima of the autocorrelation function.
- *Retinal arterial and venous coefficients of periodicity at HF and LF.* The coefficient of periodicity is the quotient of the power of the periodic component and the power of the stochastic component of an analyzed signal. For the calculation the mean amplitude M_med_ of the autocorrelogram is computed as the median of all intervals (M) between neighboring local maxima and minima of the autocorrelation function. The mean amplitude of the periodic component is calculated as half of M_med_: M_period_ = M_med_/2. The amplitude of the stochastic component of an analyzed signal is determined as the difference between the value of the autocorrelation function at lag T = 0 and the mean amplitude of the periodic component: M_stoch_ = AF(0)-M_period_ The periodicity of the signal is characterized with the coefficient of periodicity as follows K_period_ = M_period_/M_stoch_.

### Assessment of spatial arterial blood column diameter change (the longitudinal vessel profile)

**Fig. 2:**
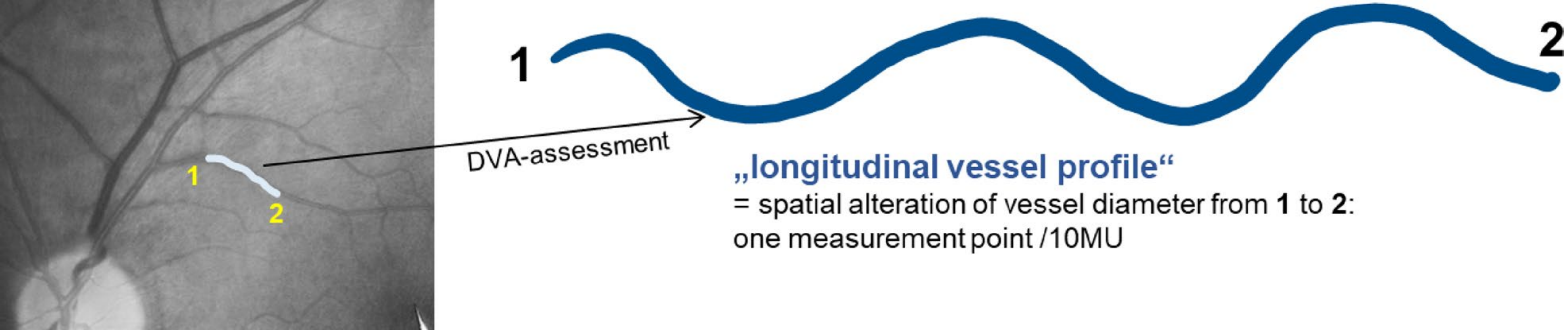
Visualization of the longitudinal vessel profile as spatial diameter changes of the retinal arterial/venous blood column along its longitudinal section. If we assume that a vessel is symmetrical to its longitudinal axis, the longitudinal vessel profile would resemble the longitudinal configuration of the inner vessel wall.

Data assessment with DVA allows the observation of spatial changes in vessel blood column diameter along a selected retinal vessel segment at designated intervals^13,24^. Through this feature it is possible to analyze non-invasively in-vivo dynamic variations in the vessel longitudinal structure in humans at different stages of a vessel reaction. Differences in diameter along the vessel segment during a defined time period can be measured. The method of data acquisition for spatial vessel analysis with DVA was explained in detail previously^18,19^. For each position along a measured vessel segment the mean of all measurements in this location during the chosen time interval is calculated. We termed the result “longitudinal vessel profile” (Fig. 2). Longitudinal arterial and venous profiles obtained at different time intervals to the peculiar stages of vessel reaction can be compared^18,19^. In DVA the measured vessel segments are scanned along with a sampling rate of 1 measuring point/12.5 MU and then interpolated to a more suitable rate of 1 measuring point/10 MU.

From each 350-s DVA examination, the first flicker stimulation cycle (out of three assessed) was selected for evaluation as the basis for our spatial analysis. It started 20 s after the start of the examination and consisted of 30 s baseline, 20 s flicker stimulation and 80 s observation period. For the arteries, five time intervals of 5 s each were defined for the spatial analysis:

1. Baseline (just before the start of flicker stimulation): 26–30 s of the cycle
2. Dilation 1 (during flicker stimulation, just before it ends): 46–50 s of the cycle
3. Dilation 2 (immediately after the end of the flicker) 50–54 s of the cycle
4. Constriction (5 s individually in the interval 55–110 s of the cycle)
5. Restoration (at the end of the cycle) 126–130 s of the cycle

For each subject, longitudinal vessel profiles were evaluated at the defined time intervals during the selected time segments. The start of time segment (4) was assigned on an individual basis. This individual time interval included the point of maximum constriction for each subject. Due to the peculiar shape of the temporal flicker response in veins, intervals (4) and (5) were defined slightly differently for veins than for arteries:

4) Recovery 1 (some time after cessation of stimulation) 86–90 s of the cycle
5) Recovery 2 (at the end of the cycle): 126–130 s of the cycle

To analyze the spatial frequency distribution within the longitudinal profiles, we obtained power spectra by means of Fast Fourier Transformation. Each power spectrum was reduced by dividing each value in the frequency distribution by the whole area of the power spectrum. Subsequently, areas under the curve (AUC) of the power spectrum within the frequency bands of interest were calculated for each subject and vessel type. For each vessel type and for each examined group, average power spectra (ARPS) were derived from those reduced individual power spectra by calculation of the median value in the group for each point of frequency distribution. From the visual assessment of individual longitudinal profiles in Fabry patients and in healthy controls, representative spatial waves of certain frequencies were assessed in both groups. The corresponding spatial frequency ranges were then derived, and the power spectra were used to check whether the effects were statistically confirmed for both groups.

### Approximate estimate of the sample sizes

Only a few Fabry patients were available for this pilot study, due to the rarity of this lysosomal storage disease in the general population. However, because of the strong effects on dynamic retinal vessel behavior observed in the first DVA measurements in Fabry disease, we designed a pilot study with a relatively small sample size of n = 10. An approximate a priori sample size calculation for the crucial parameters of the multimodal dynamic retinal vessel analysis approach in this study showed us that we might have enough power to compare the whole groups and might be underpowered in case of subgroup formation. The numbers for the power calculation were taken from the first measurements of Fabry patients and from our previous studies.^10,12,29,30^. In order to maintain the power 1−ß = 0.8 at α = 0.05 in the pairwise comparison of the subgroups, we used a simplified formula for the sample size n in each group, while comparing means with two-sample t-test^31^: n = 16 ∗ (σ/δ)^2^.

- Mean maximal vessel dilation [% to baseline]: to detect the difference δ = 2% between groups with a standard deviation of σ = 1.5% (results in effect size of 1.3), n = 9 per group is required.
- Mean venous period at low frequencies (LF), [s]: to detect the difference δ = 18 s between groups with a standard deviation of σ = 12 s (results in effect size of 1.5), n = 7 per group is required.
- AUC under ARPS of longitudinal arterial profiles 0.1–0.2 Hz, [non-dimens.]: to detect the difference δ = 0.200 between groups with a standard deviation of σ = 0.100 (results in effect size of 2), n = 4 per group is required.

### Further statistical analysis

Descriptive statistics were given by median and interquartile range. Group comparisons were performed using Mann–Whitney-U test. All the comparisons in this pilot study were carried out exploratively without correction for multiple comparisons in order to show tendencies of the different vessel behavior in the groups and to suggest additional candidates for biomarkers characterizing retinal vascular alterations in Fabry’s disease. All statistical hypotheses’ testing was conducted on two-sided 5% significance levels. Statistical analysis was performed using SPSS v.26 (IBM, Armonk, USA).

## Results

### Examination of the arterial and venous response to flicker light

**Fig. 3:**
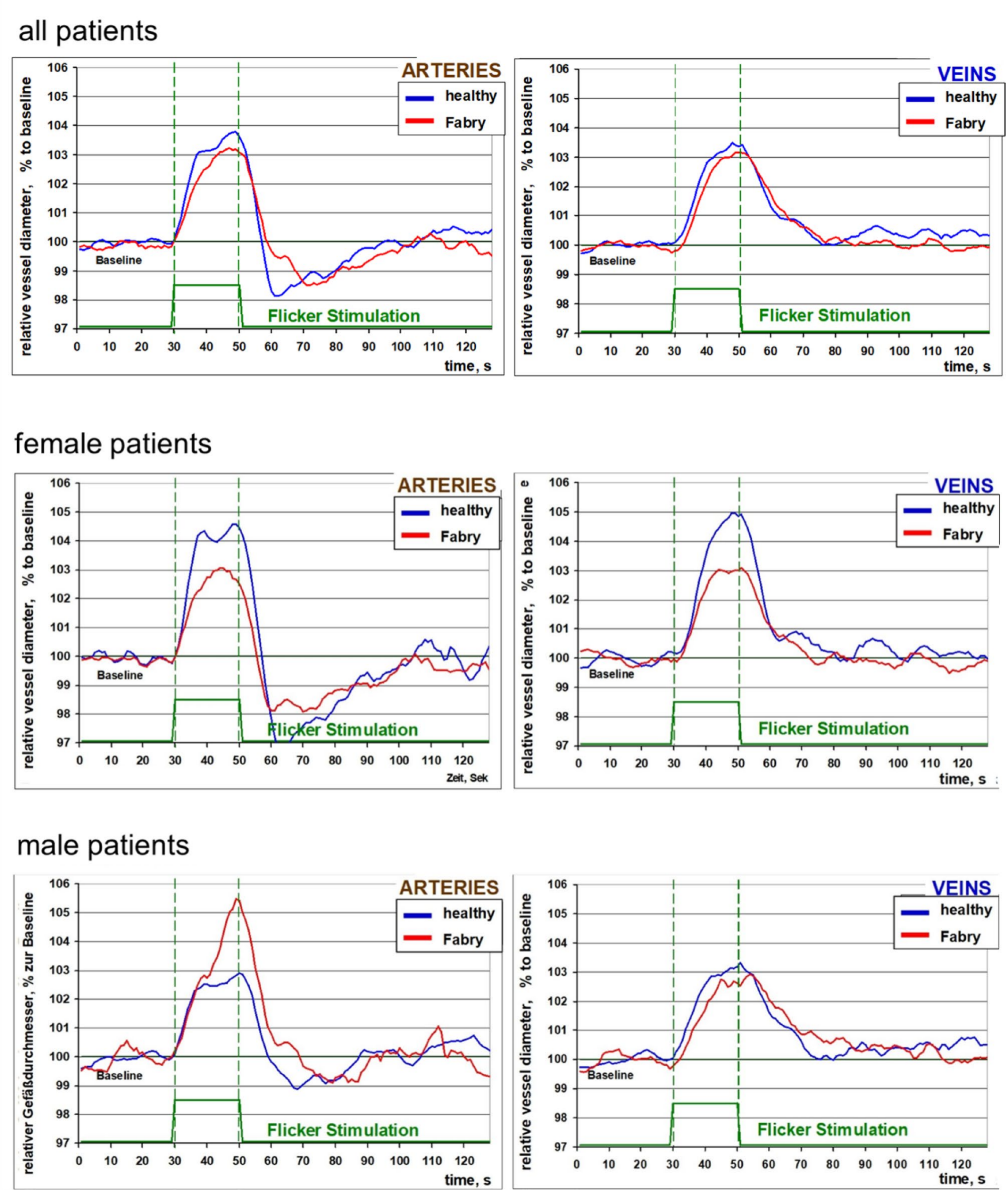
Averaged reaction to flickering light in the Fabry’s disease group. Top: whole group (n = 10); middle: female patients (n = 4); bottom: male patients without the child (n = 5).

The results of the reaction of retinal arteries and veins to flicker stimulation are presented in the Table 1 and Fig. 3. The entire patient group did not show a significant difference in the reaction to the flicker light compared to the control group (Fig. 3, upper panels). After dividing the patient group by gender, male patients with Fabry’s disease showed a significantly stronger arterial dilation of 6.3% (5.6–7.9%) compared to age-matched control participants (3.3% (2.5%–4.1%)), *p* < 0.01. The arterial peak values were also significantly higher in the Fabry male patients (*p* < 0.05). The venous responses in this subgroup were similar to those of the male participants in the control subgroup (Fig. 3, bottom panels). In contrast, women showed a reduced arterial response of 3.4% (3.1–3.7%) compared to age-matched healthy participants (5.4% (4.0–6.7%)) (*p* = 0.1, Fig. 3, middle panels). The arterial peak values were significantly reduced as compared with the control female participants in the subgroup: 6.4% (5.9–6.7%) vs. 9.0% (7.7–10.6%), *p* < 0.05. (Fig. 3, middle panels). The venous dilation in the subgroup of female Fabry patients seems to be reduced as well, however the result was not significant. The young male with Fabry’s disease showed relatively large arterial diameter, reduced arterial dilation and arterial peak value and relatively large venous dilation (Table 1).

**Table 1:** Values of parameters of retinal vessel reaction to flickering light and spontaneous retinal vessel oscillations, presented as: median (1st quartile–3rd quartile).

Parameter/group	Whole groups (n = 10)		male patients (n = 5)		Female patients (n = 4)		Male child
	Fabry	control	Fabry	control	Fabry	control	Fabry
Arterial diameter, (MU)	106.3 (95.6–115.9)	115.7 (106.4–123.0)	95.4 (83.8–112.6)	113.9 (108.1–119.9)	111.5 (104.1–119.8)	111.6 (105.1–119.6)	132.6
Mean maximal arterial dilation, (% baseline)	4.6 (3.4–6.2)	4.0 (2.8–4.6)	6.3 (5.6–7.9)**	3.3 (2.5–4.1)	3.4 (3.1–3.7)	5.4 (4.0–6.7)	1.1
Arterial peak value, (% baseline)	6.8 (6.3–9.8)	7.1 (5.0–9.7)	10.1 (8.7–10.4)*	5.2 (3.7–6.3)	6.4 (5.9–6.7)*	9.0 (7.7–10.6)	4.1
Mean maximal arterial constriction, (% baseline)	− 2.8 (− 3.2 to − 2.2)	− 2.3 (− 4.0 to − 1.1)	− 2.3 (− 4.0 to − 1.3)	− 1.9 (− 2.4 to − 1.1)	− 2.9 (− 3.1 to − 2.6)	− 3.9 (− 4.8 to − 3.0)	− 3.0
Venous diameter, (MU)	151.7 (139.0–157.0)	147.5 (138.0–154.8)	147.2 (132.2–165.1)	147.5 (139.2–156.2)	153.0 (147.3–155.7)	142.5 (136.8–148.2)	152.4
Mean maximal venous dilation, (% baseline)	3.5 (3.0–4.1)	3.5 (2.9–4.9)	3.8 (2.9–5.0)	2.9 (2.7–3.6)	3.2 (3.1–3.5)	5.7 (4.4–6.7)	5.4
Mean arterial period: HF, (s)	1.08 (0.90–1.17)	1.07 (0.95–1.11)	1.15 (0.72–1.16)	1.03 (1.03–1.08)	1.08 (1.00–1.22)	1.10 (1.05–1.15)	0.88
Mean venous period: HF, (s)	1.10 (0.92–1.31)	1.07 (0.85–1.08)	1.16 (0.74–1.35)	1.08 (0.85–1.08)	1.07 (1.02–1.22)	1.08 (1.02–1.16)	0.88
Mean arterial period: LF, (s)	11.0 (10.0–13.8)	12.7 (7.7–14.6)	13.8 (8.1–14.2)	13.8 (13.0–15.0)	10.5 (10.2–10.9)	6.1 (2.3–10.5)	13.4
Mean venous period: LF, (s)	29.9 (16.5–40.0)*	12.0 (7.7–15.5)	16.5 (16.5–40.0)	14.5 (10.1–15.1)	30.0 (18.5–40.0)	4.9 (2.0–15.0)	16.6
Arterial coeff. of periodicity HF			0.065 (0.048–0.070)	0.065 (0.050–0.080)	0.140 (0.120–0.165)	0.070 (0.055–0.090)	0.033
Venous coeff. of periodicity HF			0.048 (0.038–0.051)*	0.154 (0.135–0.154)	0.027 (0.020–0.325)	0.145 (0.105–0.180)	0.024
Arterial coeff. of periodicity LF			1.010 (0.580–1.207)	1.610 (1.602–2.435)	1.420 (1.205–1.710)	0.610 (0.085–1.303)	0.972
Venous coeff. of periodicity LF			1.310 (1.310–1.590)	1.315 (1.250–1.910)	1.820 (1.689–1.902)*	0.760 (0.218–1.344)	1.513

Significance for continuous variables (Mann–Whitney-U-test) without correction for multiple comparisons: *p < 0.05; **p < 0.01. 1 MU corresponds to 1 µm in Gullstrand’s eye model. *HF* high-frequency oscillations related to heart frequency (period < 1.5 s); *LF* low-frequency oscillations related to vasomotions (period ≥ 1.5 s).

### Assessment of oscillatory temporal changes of retinal vessel diameter

**Fig. 4:**
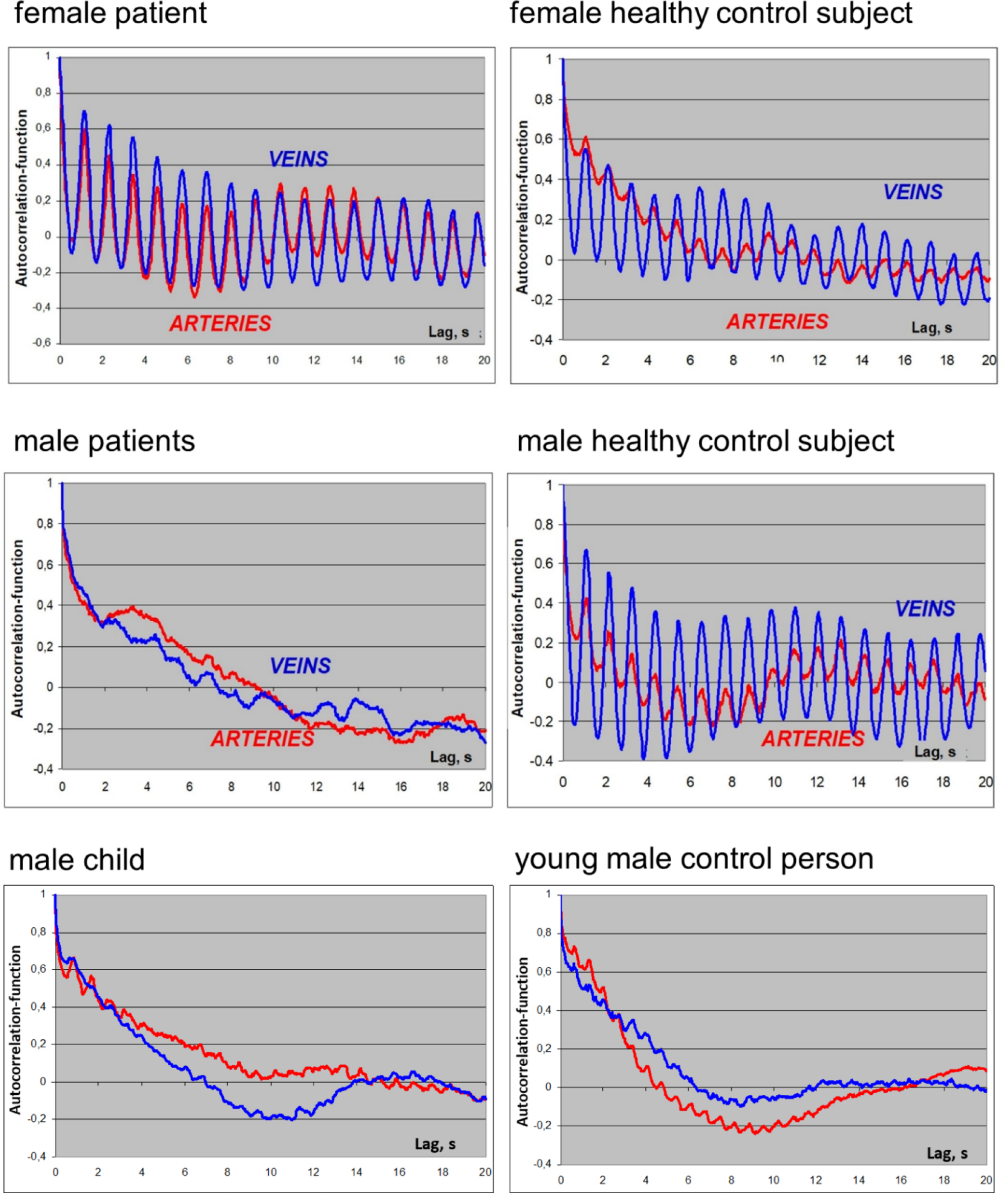
Characteristic examples of autocorrelation functions in the examined groups. Left column: patients with Fabry’s disease; right column: control group. Top: female patients; middle: male patients; bottom: young male patient.

In the analysis of the periodicity of spontaneous, unstimulated vascular wall modulation we found differences in retinal vessel behavior of Fabry patients (Table 1, Fig. 4). Due to the small sample size and therefore relatively low statistical power, especially after splitting into gender subgroups, we focus on the most prominent findings in terms of effect size. (Table 1). The mentioned aspects of spontaneous retinal vessel behavior were rather different in the male and female subgroup of Fabry patients. The female subgroup shows in comparison to the corresponding control subgroup:

- Equal periods of cardiac rhythm
- Less scattered and longer periods of LF oscillations in arteries (not significant)
- Longer LF periods in veins (not significant)
- Higher HF periodicity in arteries and veins (not significant)
- Higher LF periodicity in arteries (not significant)
- Higher and less scattered LF periodicity in veins (significant)

The male subgroup of Fabry patients shows in comparison to the corresponding controls:

- Equal periods of cardiac rhythm (larger variation)
- Equal and more scattered LF periods in arteries
- Longer and more scattered LF periods in veins (not significant)
- Lower LF periodicity in arteries (not significant)
- Lower HF periodicity in veins (significant)

From the quantitative data and the visual analysis of autocorrelograms it seems that spontaneous unstimulated retinal vessel behavior is altered mostly dramatically in male Fabry patients (Table 1; Fig. 4, middle panel) with overall less periodic and more chaotic arterial and venous oscillations within the studied time frame. Confirming this observation, the quantitative data summarized in Table 1 show statistically significant changes in venous HF periodicity in Fabry male patients and a notable difference in their arterial LF periodicity that did not reach statistical significance. Meanwhile, the value of the venous period at low frequencies differs between Fabry patients and healthy participants, and it seems that female patients mainly contribute to this difference (Table 1). The autocorrelation patterns and the corresponding parameters of the male child did not differ essentially from those of the young control person (Table 1; Fig. 4 lower panel).

### Assessment of spatial arterial blood column diameter change (the longitudinal vessel profile)

**Fig. 5:**
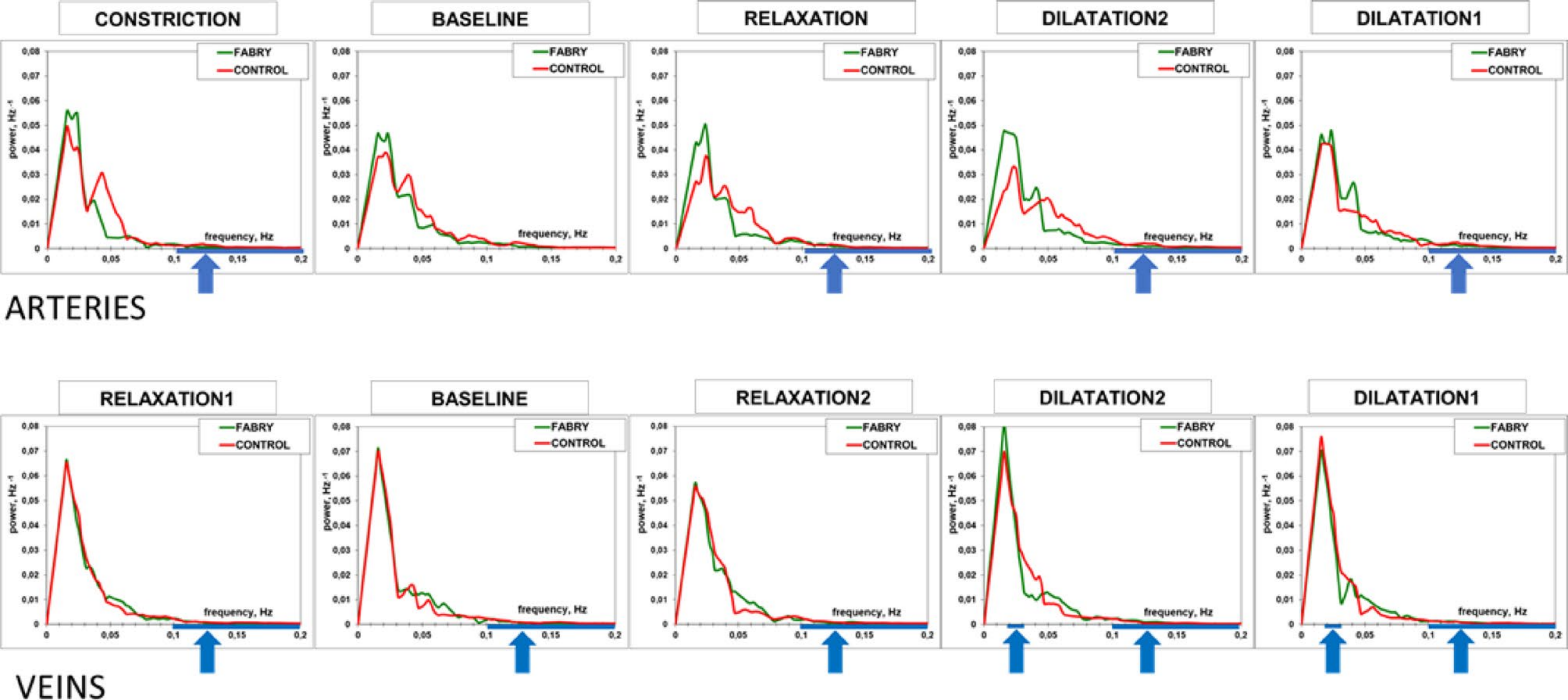
Average reduced power spectra of spatial longitudinal arterial (top panel) and venous (bottom panel) profiles at the 5 phases of vessel reaction during flicker stimulation assessment—summary for the whole groups. The sequence of phases was chosen according to the increase in vessel diameter from the left to the right. Blue arrows and lines show spatial frequency ranges, where the area under the power spectra curves was significantly different between the groups (Table 2; p < 0.05). This means a more irregular arterial blood column during the arterial response (functional alteration) in the Fabry group and a more irregular venous blood column (structural alteration) with some additional patterns during venous dilation as additional functional alterations in the Fabry group.

**Fig. 6:**
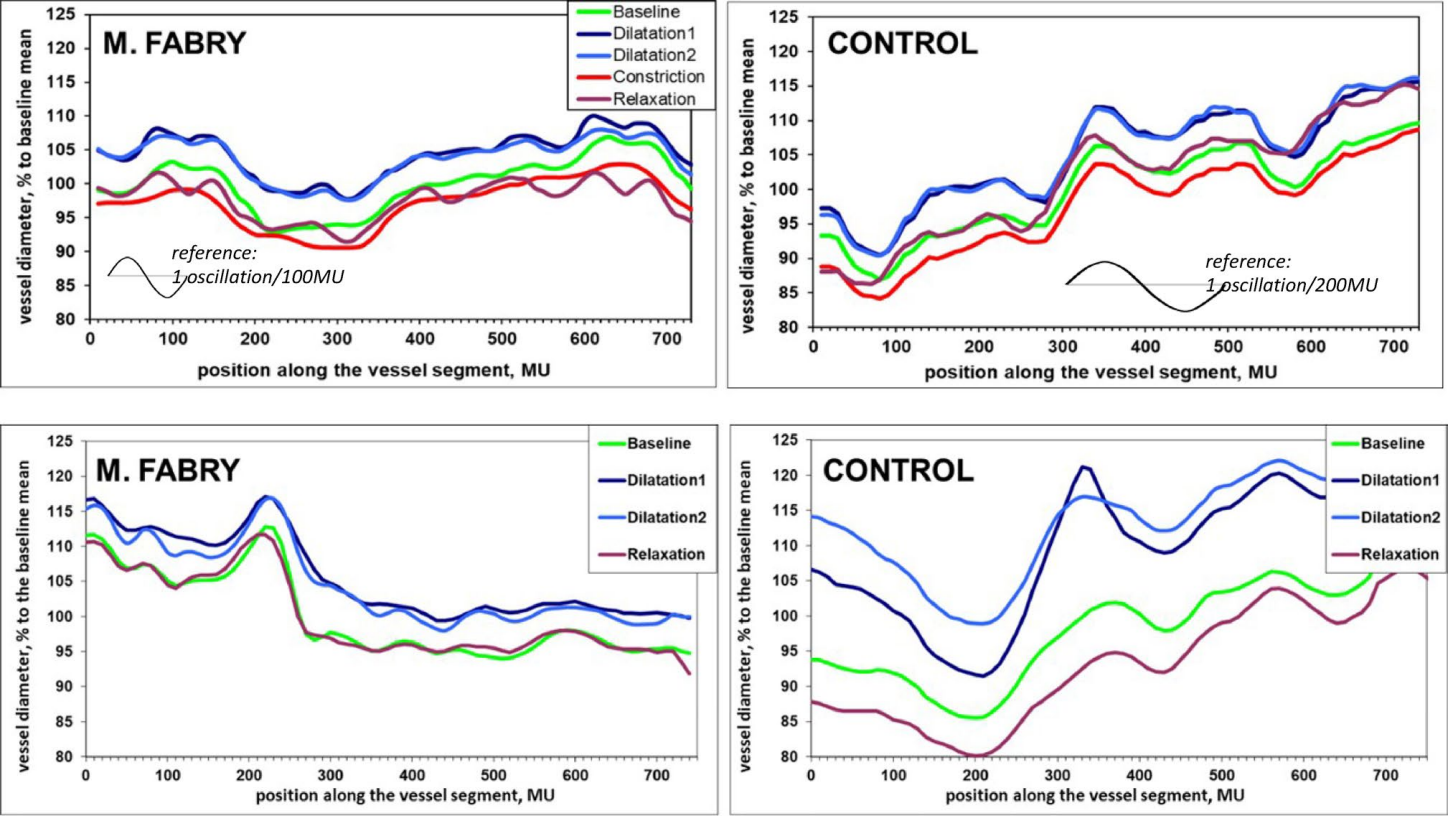
Characteristic examples of longitudinal retinal vessel profiles in compared groups at different phases of the vascular response to flicker. Top: arterial profiles. Notice a characteristic irregular structure of arterial profiles in M.Fabry, that possess periods of ~ 70–100 MU and slightly smother profiles at the baseline and constriction. The control person shows small irregularities with much smaller periods and larger ones with periods of ~ 200 MU. Bottom: venous profiles. Notice quite an irregular structure of the longitudinal profiles in the Fabry patient with periods of ~ 50–100 MU in all phases of the venous reaction, compared with smoother lines of the profiles in the control person. 1 MU corresponds to 1 µm in Gullstrand’s eye model.

The longitudinal microstructure of retinal arteries and veins in Fabry’s disease was structurally and functionally changed. Group ARPS of spatial longitudinal arterial and venous profiles at the 5 stages of vessel reaction during flicker stimulation assessment are presented in Fig. 5. Values of quantitative parameters calculated as AUC under individual ARPS within frequency bands are summarized in Table 2. For this evaluation modality the resulting longitudinal vessel profiles were quite similar for male, young male and female patients with Fabry’s disease, so that we refused on detailed statistical evaluation in subgroups in order to show the main revealed effects with a higher statistical power. AUC within 0.1–0.2 Hz in Fabry group were larger in veins at all stages of venous reaction as well as at all stages of arterial reaction except the baseline. This resembles the presence of irregularities/additional spatial waves with periods of ~ 50–100 MU in retinal vessels of Fabry patients, as it can be seen in a characteristic example in Fig. 6, left panel. Those irregularities seem to be persistent in retinal veins, representing a structural alteration in veins in Fabry’s disease. In the meantime, retinal arteries in Fabry’s disease seem to become more irregular during the vessel reaction, while the irregularities “disappear” or better to say, were less pronounced at the baseline. In the Fig. 6, top right panel, one can see the effect, that in this case is also extended to the constriction profile. Here we might report a functional alteration: the peculiar structure of the profile is more pronounced during vessel reaction. Interestingly, the characteristic irregularities possess the same periods both in arteries and in veins in Fabry’s disease.

**Table 2:** Values of spectral parameters of longitudinal retinal vessel profiles, presented as: median (1st quartile–3rd quartile).

Parameter/group	Whole groups (n = 10)		p-value
	Fabry	Control	
AUC under ARPS of longitudinal arterial profiles 0.1–0.2 Hz, [non-dimens.]: period: ~ 50–100 MU			
Baseline	0.035 (0.026–0.072)	0.052 (0.038–0.076)	0.526
Dilation 1	0.217 (0.140–0.322)	0.061 (0.028–0.092)	0.008**
Dilation 2	0.223 (0.120–0.322)	0.062 (0.034–0.090)	0.008**
Constriction	0.157 (0.109–0.312)	0.042 (0.027–0.067)	0.016**
Relaxation	0.172 (0.116–0.331)	0.038 (0.024–0.086)	0.012**
AUC under ARPS of longitudinal venous profiles 0.1–0.2 Hz, [non-dimens.]: period: ~ 50–100 MU			
Baseline	0.172 (0.120–0.280)	0.046 (0.030–0.056)	0.006**
Dilation 1	0.150 (0.135–0.272)	0.031 (0.016–0.052)	0.002**
Dilation 2	0.172 (0.131–0.247)	0.035 (0.020–0.047)	0.002**
Relaxation 1	0.203 (0.153–0.286)	0.045 (0.027–0.054)	0.001**
Relaxation 2	0.218 (0.149–0.317)	0.044 (0.032–0.053)	0.002**
AUC under ARPS of longitudinal venous profiles 0.02–0.03 Hz, [non-dimens.]: period: ~ 333–500 MU			
Baseline	0.180 (0.163–0.198)	0.201 (0.181–0.223)	0.326
Dilation 1	0.176 (0.156–0.196)	0.221 (0.186–0.240)	0.012*
Dilation 2	0.179 (0.134–0.198)	0.214 (0.180–0.245)	0.013*
Relaxation 1	0.188 (0.163–0.206)	0.218 (0.183–0.234)	0.070
Relaxation 2	0.192 (0.160–0.215)	0.225 (0.195–0.232)	0.170

Significance for continuous variables (Mann–Whitney-U-test) without correction for multiple comparisons: *p < 0.05; **p < 0.01. *AUC* area under the curve, *ARPS* average reduced power spectra. 1 MU corresponds to 1 µm in Gullstrand’s eye model.

Additionally, we report another, presumably both functional and structural alteration, in retinal veins in Fabry’s disease. AUC within 0.02–0.03 Hz in Fabry group were smaller in veins, especially during venous dilation. This finding resembles a „disappearance” of the waves with periods of ~ 333–500 MU in Fabry patients. The longitudinal venous profiles seem to become generally flatter in Fabry’s disease (Fig. 6, bottom panels). This effect can be revealed at all stages of the venous reaction, while it was especially emphasized during dilation, showing statistical significance compared to the control group (Table 2).

In summary, the results on longitudinal retinal vessel profiles show characteristic irregularities of arterial blood column during arterial reaction (functional alteration) in Fabry’s disease group as well as the same characteristic irregularities of venous blood column (structural alteration). Additionally, longitudinal retinal venous profiles in Fabry’s disease become flatter, which is more emphasized during venous dilation.

## Discussion

Our results suggest peculiar distortions in microvascular function in Fabry’s disease which may reveal underlying pathomechanisms in vivo. As expected, since Fabry’s disease is X-linked and has more severe effects in affected males, we observed more pronounced vascular abnormalities in the male cohort.

From our results, we hypothesize that the more pronounced retinal arterial and venous responses to flickering light in male Fabry patients are associated with alterations in the regulatory components of retinal vascular responses, while a presumed arterial narrowing may also contribute to the effect. Indeed, the diameters of the retinal arterial segments examined in Fabry patients were on average smaller than those in the control group, and it seems that male patients mainly contribute to this difference (Table 1). However, we observed that the arterial constriction in the male subgroup was of the same magnitude as in the control participants, which contradicts the assumption of a general arterial constriction in male patients.

On the other hand, a contribution of sclerosis-like alterations of the retinal arterial wall in female Fabry patients can be assumed, since the peak value in this subgroup was significantly reduced, with both dilation and constriction contributing to this effect (Table 1). In addition, the tendency for veins to be wider in female Fabry patients, together with reduced venous dilation, may indicate retinal venous widening, possibly related to local inflammation.

We observed chaotic baseline vasomotions in our male Fabry patients (Fig. 4, middle panel). This reflects reduced general smooth muscle activity in the relaxed vessel wall and thus represents an imperfect equilibrium in the unstimulated vasculature, resulting in an inappropriately pronounced vessel dilation to the strongest NO-mediated stimulus for retinal vessel dilation, namely, NO-mediated vascular coupling-dependent vessel dilation due to flicker light. Moreover, another finding—the functional alteration of the retinal arterial blood column during the vessel reaction to flicker can also be interpreted as a local deficiency of smooth muscle activity within the arterial wall (Fig. 5, top panel; Fig. 6, top panel). This raises the question: where is the defect in retinal vessel reaction to flicker light in male Fabry patients? Is it sensor-, namely vascular endothelium-mediated, or rather effector-, namely smooth musculature of the arterial vessel wall-mediated? Our data, for the first time, show that the retinal vasculature in male Fabry patients, in an unstimulated baseline status, is rather chaotic in its low-frequency modulation, which represents the vasomotions. As recent research has shown, the proper function of potassium channels in the cell wall is important for the regular function of smooth musculature of vessels; however, this function is altered in Fabry’s disease^32^.

We postulate that in Fabry’s disease, the underlying mechanism leading to ischemic stroke is located in the baseline smooth muscle vessel wall regulation, as shown here in our male Fabry patients. Since this potassium channel-mediated normal reaction is especially altered in the affected male patients, a normal reaction to flickering light is not possible. The endothelium is signaling a demand for vessel diameter increase, and the unfiltered response is an abnormally large dilation, as demonstrated in our study in the affected male individuals, due to an altered effector response.

An alternative explanation of our findings is that the pronounced reaction to flicker in male Fabry’s disease patients might be related to the distortion of neuro-vascular coupling within the neuronal tissue of the retina, due to the accumulation of fats in the tissue. Similar pronounced arterial and venous reactions to flickering light have been recently reported in Alzheimer’s disease dementia^28^, whereas in other vascular-origin disorders investigated by our research group, we observed reduced retinal arterial, and sometimes venous, dilation in response to flicker^12,21,22^. Similar to Alzheimer’s disease, with its Amyloid-plaques and tau tangles in the neuronal tissue, which may reduce the functionality of the neuro-vascular unit^28^, the accumulation of glycosphingolipids may distort the neuro-vascular coupling in severe Fabry’s disease, leading to the unbalanced overemphasized flicker response in Fabry male patients. In contrast, reduced arterial and venous responses in female Fabry patients might be explained by presumed endothelial dysfunction and increased vascular stiffness in moderate Fabry’s disease^9^, making their reduced flicker reaction similar to responses seen in arterial hypertension^33^, obesity^12^, and glaucoma^20^.

As mentioned above, one proposed mechanism for the vascular dysfunction observed in Fabry’s disease is the abnormal accumulation of glycosphingolipids within endothelial cells. This accumulation may lead to smooth muscle proliferation in the arterial media layer, which can cause damage to the arterial wall and impair blood flow. Such damage may ultimately lead to stroke and other vascular complications^8,34^. We assume that we were able to reveal this aspect of microvascular dysfunction by showing peculiar irregularities within retinal vessel profiles in Fabry’s disease. It seems that the peculiar shapes of arterial and venous blood columns in Fabry’s disease, with characteristic alternating depressions and projections every 50–100 µm, along the vessels represent those patterns. These might relate to the damaged endothelial cells, sites of smooth muscle proliferation along the vessels, or accumulations within the perivascular space. The retinal veins appear to have these persistent changes, while retinal arteries, with their thicker walls and multifold muscular layers, make those patterns of the blood column visible mostly during reactive vessel wall motion.

Spontaneous non-stimulated dynamic retinal vessel behavior was altered in Fabry’s disease, with these alterations being more pronounced in male patients (Fig. 4). The heart frequency pulsations in the veins were less periodic. The low-frequency oscillations related to vessel vasomotions were less periodic in arteries and exhibited considerable scatter. This latter finding may also be explained by the pronounced accumulation of lipids in the vessel walls as well as in the perivascular space. Recently, we reported on the distorted retinal vessel vasomotions in Alzheimer’s disease dementia, which might be linked to the dysregulation of paravascular drainage and thus contribute to the amyloid clearance hypothesis^29^. Similarly, we can hypothesize that the aperiodic arterial vasomotions in male Fabry’s disease patients may represent a sign of ultimate dysregulation of the already distorted paravascular drainage of lipids. Meanwhile, the longer periods, as well as the higher periodicity of vasomotions in arteries and veins of female Fabry’s disease patients, could indicate compensatory upregulation of the designated pulsations, which probably still contribute to the removal of lipids at moderate disease stages.

This pilot study has several limitations, most notably the relatively small sample sizes. As we identified potentially gender-specific effects on retinal vessel dynamics in Fabry’s disease, subgroup analyses were performed, which further reduced the statistical power. A marked arterial dilation was observed in male patients but not in female patients. While this finding may suggest a sex-specific vascular response in Fabry disease, its interpretation remains speculative and warrants validation in larger, well-characterized cohorts.However, even in the small groups we were able to show some strong effects, that outline peculiar microvascular alterations in Fabry’s disease. The lack of thorough clinical characterization of our Fabry group is another limitation of this pilot study. Larger clinical studies with more detailed characterization and phenotypisation of Fabry patients are needed to confirm and, possibly, to better clarify the revealed findings.

In conclusion, we applied multimodal dynamic retinal vessel analysis to reveal retinal microvascular alterations in Fabry’s disease. Our findings indicate that the retinal arterial response to flicker stimuli is significantly more pronounced in male patients with Fabry’s disease, while it is reduced in female patients. Spontaneous retinal vessel oscillations are altered in Fabry’s disease, with more dramatic changes observed in male patients, who exhibit less periodic vessel behavior over a broad frequency range. Both arterial and venous retinal vessel walls in Fabry’s disease show distinct microstructural changes, with functional irregularities in arteries and structural changes in veins.

The application of DVA represents a practical and non-invasive method for monitoring the central microvascular status in Fabry’s disease. By providing detailed insights into sex-specific vascular responses, DVA could assist in early detection of microvascular dysfunction and help guide personalized treatment decisions. Additionally, DVA may serve as a valuable tool in assessing the efficacy of potential therapies targeting underlying mechanisms, such as potassium channel dysfunction, and tracking disease progression over time. This approach could be further integrated into clinical practice to improve patient outcomes by offering more targeted monitoring and intervention strategies.

## Data Availability

The datasets generated and analyzed during the pilot study are not publicly available. However, a de-identified and anonymized version of the data may be made available from the corresponding author on reasonable request. Restrictions may apply to the availability of these data, and interested researchers should contact il@chiemseeaugentagesklinik.de for data access and further inquiries.
